# 2D functionalized copper(ii)-carboxylate framework for efficient chemical fixation of carbon dioxide: insights from experimental and theoretical analysis

**DOI:** 10.1039/d5ra04781a

**Published:** 2025-09-22

**Authors:** H. M. Kashif Javaid, Zafar A. K. Khattak, Rizwan Ashraf, Adnan Ali Khan, Yaqoob Shah, Asim Mansha, Misbah Zia, Hussein A. Younus, Nazir Ahmad

**Affiliations:** a Department of Chemistry, GC University Lahore Lahore-54000 Pakistan dr.nazirahmad@gcu.edu.pk; b Department of Chemistry, Faculty of Science, Fayoum University Fayoum 63514 Egypt hay00@fayoum.edu.eg; c Nanotechnology Research Center, Sultan Qaboos University P. O. Box 17, 123, Al-Khoud Oman; d Department of Chemistry, University of Buner Swari, Buner Pakistan; e Department of Chemistry, University of Agriculture Faisalabad Pakistan; f School of Science, Harbin Institute of Technology Shenzhen 518055 China; g Department of Applied Sciences, National Textile University Faisalabad 37610 Pakistan; h Department of Chemistry, Government College University Faisalabad Faisalabad 38000 Pakistan; i Institute of Chemistry, University of Sargodha Sargodha-40100 Pakistan

## Abstract

Metal–organic materials are an important class of heterogeneous catalytic materials that offer a variety of organic transformations. A 2D azide functionalized copper(ii)-carboxylate framework (FCCF) was synthesized for catalytic application in the chemical fixation of carbon dioxide. The synthesized MOF was characterized using different techniques, *i.e.*, FTIR, PXRD, SEM and TGA. Hirshfeld surface analysis was performed to assess intermolecular interactions. The catalytic potential of the FCCF for CO_2_ fixation to synthesize organic cyclic carbonates was investigated and optimized under solvent-free conditions and ambient CO_2_ pressure at 100 °C within 3–8 hours in the presence of a co-catalyst (tetrabutyl ammonium bromide). The complete conversion of epichlorohydrin into its cyclic carbonate with maximum selectivity was achieved under optimal reaction conditions. The FCCF as a functionalized material exhibited efficient fixation of carbon dioxide. The reaction mechanism for the cycloaddition of CO_2_ to epoxide catalyzed by the FCCF was investigated in detail based on experimental inferences and corroborated with the periodic calculations of density functional theory (DFT). Energy calculations depict that the azide-functionalized MOF material (FCCF) efficiently converts CO_2_ and epoxides into targeted cyclic carbonates. Therefore, the FCCF is an interesting material for the chemical fixation of CO_2_ for developing value-added chemical products.

## Abstract 2

A 2D functionalized Cu(ii)-carboxylate MOF is analyzed for heterogeneous catalytic fixation of CO_2_ to synthesize precious cyclic carbonates. The DFT reaction mechanism is also investigated to highlight the additional role of azide functional group.

## Graphical abstract

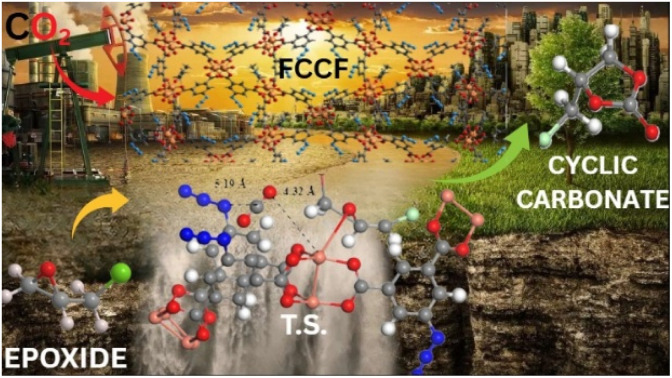

## Introduction

One of the main participants in global warming and the reduction of fossil carbon resources is anthropogenic carbon dioxide (CO_2_); this necessitates the conversion of CO_2_ into renewable bio-based raw materials.^1–3^ The rapid growth of population will intensify the overlap between industrial and residential zones.^4^ With the increasing human activities, CO_2_ emissions, primarily from biomass, power plants, vehicle exhaust, industrial processes, and fuel combustion, will significantly contribute to natural disasters, such as ecosystem disruption, sea level rise, species extinction, crop failure, and drastic environmental imbalance *etc.*^5^ To reduce its emissions, carbon dioxide can be captured, stored, and used for its conversion into value-added products. Moreover, carbon dioxide may be used in CO_2_ cycloaddition through an epoxide, finally resulting in splendid cyclic carbonates, such as ethylene carbonate, propylene carbonate, and dimethyl carbonates *etc.*^6^ These targeted cyclic carbonates have extensive utilities in industries. They are used as electrolytes in Li-ion batteries, lubricants, solvents, pharmaceuticals, and also building blocks in the synthesis of polymers.^7–9^ Over the years, countless catalysts have been employed for CO_2_ fixation, but they frequently suffer from disadvantages such as high catalyst loading, low yields, poor recyclability, and harsh conditions. Therefore, designing stable, efficient, and eco-friendly catalysts is a highly demanded global necessity.^10^ Metal complexes and metal salts have also been used effectively for CO_2_ utilization reactions.^11–15^ Metal–organic frameworks (MOFs) are highly porous and crystalline materials with characteristics such as high surface area, robustness, and extraordinary adsorption capacity and can be directly synthesized by appropriate methodologies, making them fitting materials for catalysis.^16–19^

In comparison to other porous materials, such as mesoporous silica and zeolites, MOFs offer even catalytic sites and improved chemical manageability, enhancing product selectivity. Active metal sites can be incorporated into MOF pores to generate frameworks with high metal density and bulky apertures.^20,21^ The flexibility in the organization of active centers such as metallic nodes and organic linkers, in addition to their chemical synthetic functionalization, demonstrates the potential of particularly modified MOFs for intended catalytic challenges.^22^ These features enable MOF materials to outshine in their advanced catalytic applications in organic transformations such as CO_2_ addition into epoxides. Heterogeneous MOFs catalysing epoxide-CO_2_ transformations under mild conditions allow easy separation and reuse for several catalytic runs. The cyclic carbonate synthesis through CO_2_ and epoxides using MOFs with suitable co-catalysts is an efficient catalytic system.^23^ Different MOFs, *i.e.*, Co(ii), Cu(ii)-, Zn(ii)-, and Cd(ii)-based MOFs entailing derivatives of benzene-dicarboxylic acid and amide functionality have been reported for CO_2_ fixation.^24–26^ In the present study, a functionalized copper(ii)-carboxylate framework (FCCF) has been synthesized^27^ and is investigated for the fixation of carbon dioxide (CO_2_) to organic cyclic carbonates.

## Results and discussion

**Fig. 1:**
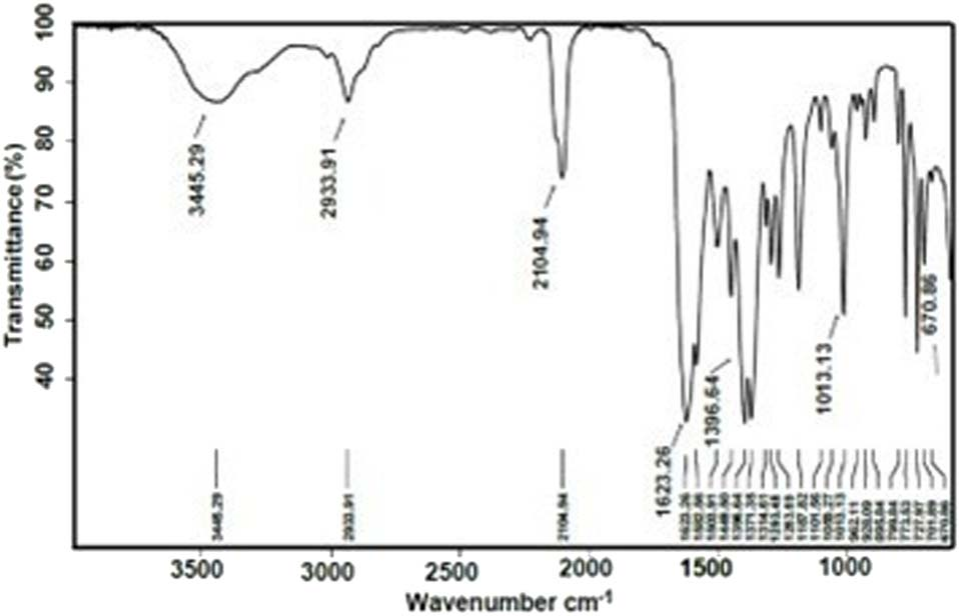
FTIR spectrum of the as-synthesized FCCF.

**Fig. 2:**
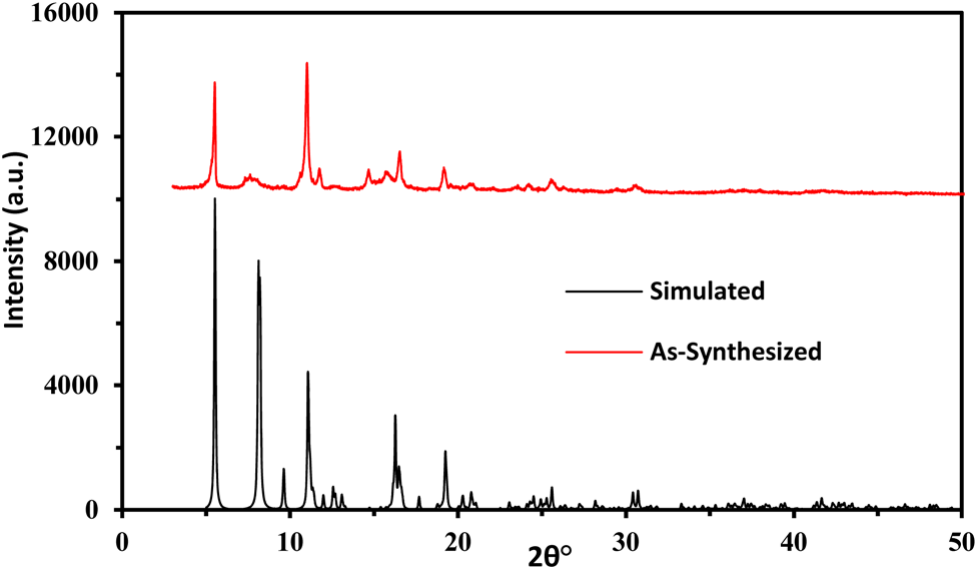
Powder XRD spectrum of the simulated (reproduced from ref. 27 with permission from the Royal Society of Chemistry) *vs.* as-synthesized FCCF.

**Fig. 3:**
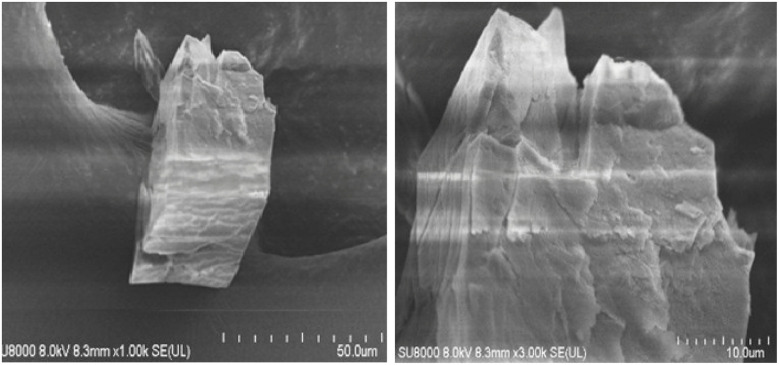
SEM images of FCCF at different magnifications.

**Fig. 4:**
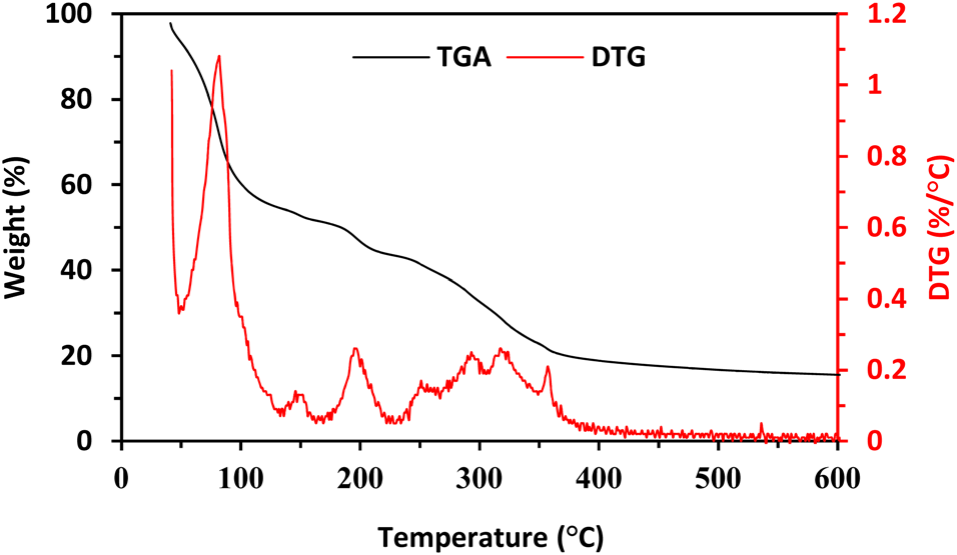
Thermal analysis (TG and derivative-TG) of the FCCF.

A functionalized copper(ii)-carboxylate framework (FCCF) has been crystallized by reacting copper salt and azide isophthalic acid in *N*,*N*′-dimethylacetamide at room temperature. The FTIR analysis (Fig. 1) of the resulted greenish blue block crystals of FCCF confirms the successful synthesis, by the presence of distinguished peaks for functional groups like azide, coordinated carbonyl with copper(ii) are identified, *i.e.*, characteristic of FCCF. There is a distinct peak at 2105 cm^−1^ corresponding to the azide (–N_3_) functional group. A sharp characteristic peak for coordinated carbonyl functionality in FCCF appeared at 1623 cm^−1^. An absorption band at 671 cm^−1^ revealed the bonding of copper to oxygen in the FCCF material. The X-ray diffraction analysis of the crystalline FCCF is performed to check the bulk phase purity. The as-synthesized pattern shows peaks that closely match the simulated pattern, verifying the successful synthesis of the copper(ii)-isophthalate framework (Fig. 2). The sharp and intense peaks well matching with the simulated pattern (black)^27^ revealed the crystalline structure and bulk phase purity of FCCF. The shape and the surface morphology of the synthesized FCCF were analysed by scanning electron microscopy (SEM). The images of FCCF crystalline material at different magnifications (as shown in Fig. 3) display a blocky angular particle (of average size dimension ∼35 μm) with definite edges and relatively flat faceted surface characteristics of a crystalline material of block shape. The surface is rough and layered like a cleaved crystalline structure having a smooth texture in contrast to sharp edges. The thermal stability of FCCF is investigated by thermogravimetric analysis (TGA) under an argon atmosphere (as shown in Fig. 4). The staged solvent molecules resulted in the weight loss of the compound up to 115 °C temperature, after which FCCF showed intact structural stability in the range of 115–193 °C. About 5% weight loss is observed in the range of 194–220 °C which could be attributed to the initiation of thermal degradation of the linker (azidoisophthalate group). The MOF exhibited stability in the temperature range of 221–243 °C and then gradual decomposition of the main linker body alongside complete structural disintegration with weight loss of about 27% in the temperature ranging from 244 to 520 °C. The further exposure of heat instigated no considerable loss of weight up to 600 °C. The remaining residual material, about 16%, is anticipated to be leftover carbonaceous material along with metallic oxide.

**Fig. 5:**
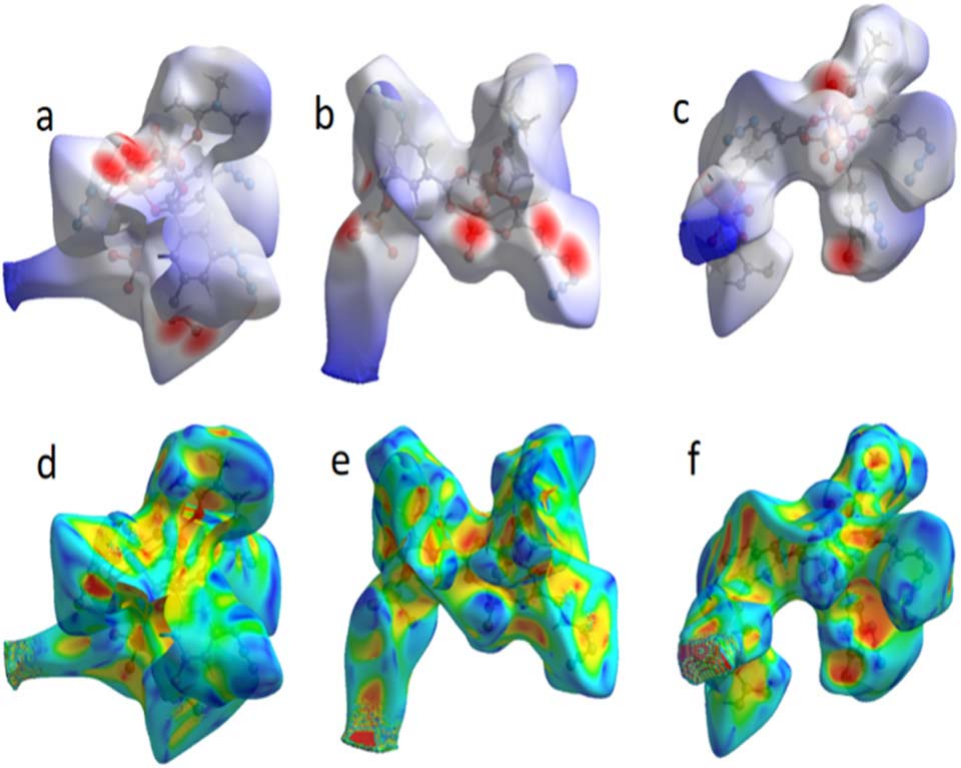
Hirshfeld surface plotted over *d*_norm_ (a–c) for the FCCF in the range −1.2204 to −7.3632 a.u.; Hirshfeld surface plotted over the shape index (d–f) in the range from −1 to 1 a.u.

To determine the intermolecular interactions in the FCCF crystal system, Hirshfeld surface (HS) analysis is employed,^28,29^ and the long- and short-range interactions in the FCCF material are differentiated.^30^ The Hirshfeld surface analysis is performed using the crystallographic information file (cif) of CCDC no. 1423822.^27^ The HS for FCCF are plotted over *d*_norm_ (normalized distances) and over shape index as shown in Fig. 5 (Fig. 5a–c and d–f, respectively), and the characteristic intermolecular interactions are represented. The non-covalent interactions in FCCF along with valuations of the interatomic H-bonding interactions are calculated by applying CrystalExplorer software (version 21.5) As shown in Fig. 5, red spots represent the short contacts among the atoms, blue spots correspond to long associations and van der Waals radii are illustrated in white color on the Hirshfeld surface. The intense blue spot in the vicinity of the Cu(ii) metallic center (as shown in Fig. 5c) illustrates a long interaction site with lower electronic density and structural strain, offering an electropositive region with strong interaction potential for electronically rich moieties, *i.e.*, oxygen atoms of both CO_2_ and epoxide molecules. Furthermore, a deep red spot around the oxygen atom of the azidoisophthalate group indicates that these atoms have strong interaction potential towards symmetry-related Cu-atoms and a latent platform in FCCF, providing a stimulating reason to investigate the catalytic potential of FCCF.

Plotting HS over the shape index is very helpful in understanding the weak interactions like π⋯π stacking. Blue and red colored triangular shape connecting patches nearby the aromatic ring displayed the presence of π⋯π interactions in the crystal structure (Fig. 5d–f).

**Fig. 6:**
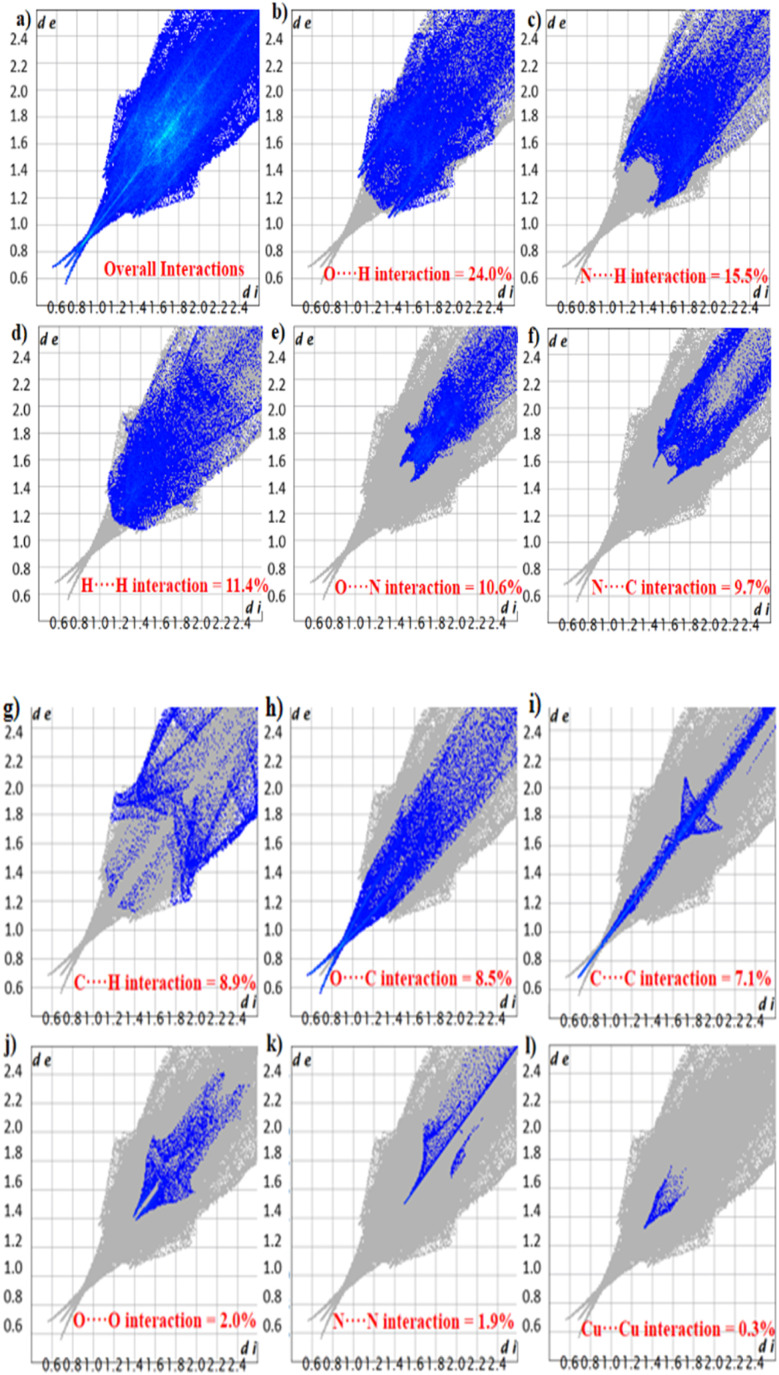
2D fingerprint plots of the FCCF for (a) overall interactions and (b–l) individual interatomic contacts with percentage contributions in the crystal packing greater than 0.1%.

2D fingerprint plot analysis helps to identify the supramolecular assembly by breaking inclusive interactions into individual interatomic interactions in the single crystal structure, including reciprocal contacts.^31,32^Fig. 6a shows the overall interaction in FCCF *via* a 2D plot. The most influencing factor in FCCF packing is O⋯H with a 24.0% contribution (Fig. 6b), alongside N⋯H having a percentage contribution of 15.5% (Fig. 6c). The lasting important interaction in FCCF is H⋯H, O⋯N, N⋯C, C⋯H, O⋯C, C⋯C, O⋯O, N⋯N, and Cu⋯Cu with contributions of 11.4, 10.6, 9.7, 8.9, 8.5, 7.1, 2.0, 1.9, and 0.3%, respectively, as shown in Fig. 6d–l. The percentage (%) contributions of the interactions revealed in FCCF are summarized in Table S1.

**Fig. 7:**
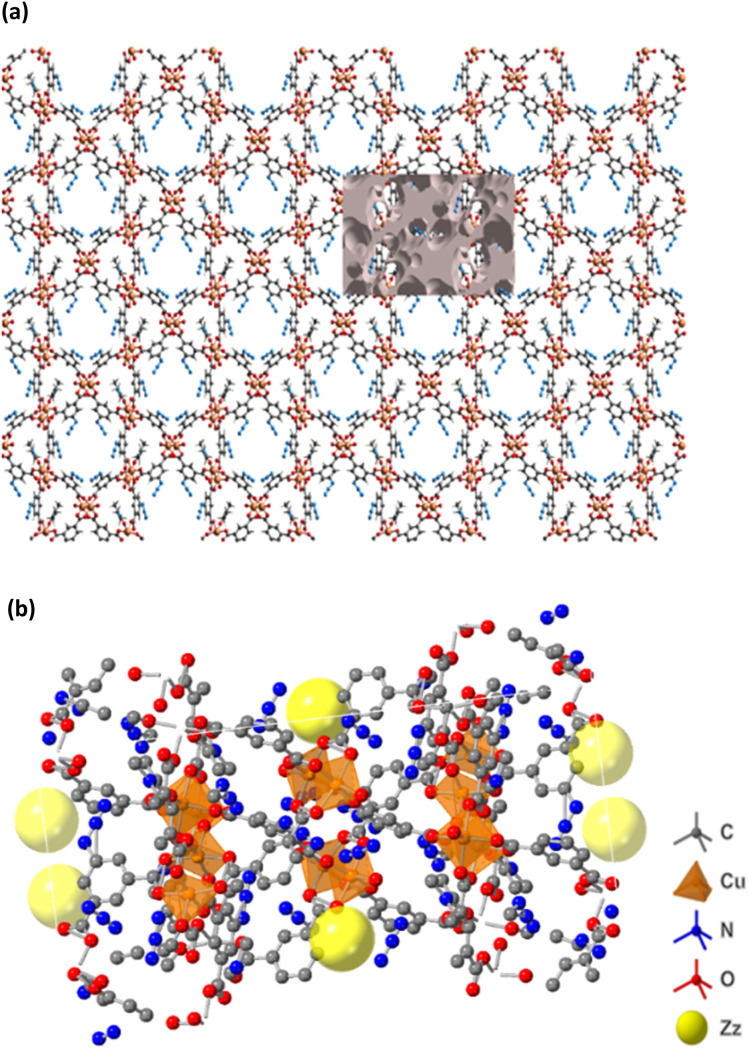
(a) Graphical representation of voids for the FCCF. (b) Dummy yellow spheres Zz as cavities.

The supramolecular arrangement *i.e.*, self-assembling into larger polymeric structure of the FCCF can be studied *via* atom-ALL and ALL-atom interactions.^33,34^ Atom-ALL represents interactions between a specific atom in the HS and all surrounding molecules, while ALL-atom involves all HS atoms interacting with a nearby surrounding atom outside the HS. The strongest interactions involve H-atoms, with H-ALL and ALL-H contributing 32.2 and 39%, respectively. The remaining interactions contributing to the crystal packing of FCCF are displayed in Fig. S1a and b. Voids play a crucial role in crystal packing, as they provide insights into single-crystal properties.^34–36^ In FCCF, voids are calculated assuming spherical symmetry and using Hartree–Fock theory, yielding a void volume of 2853.53 Å^3^ and a unit cell volume of 8661.84 Å^3^. Voids occupy 32.9% of the crystal space, indicating a porous structure with visible cavities (Fig. 7a) and offering an exciting insight about the conceivable consumption of the higher surface area in FCCF for the aimed catalytic application (fixation of CO_2_ with epoxies). To further analyse these cavities, Crystalmaker 10.7.3 software is used,^35^ by representing cavity centers with dummy atom Zz, which revealed an average cavity size of ∼4.5 Å^3^ in FCCF (Fig. 7b).

The catalytic potential of the synthesized FCCF was investigated for the cycloaddition of epichlorohydrin (ECH) to carbon dioxide (CO_2_) for the catalytic synthesis of its organic cyclic carbonate. Tetrabutyl ammonium bromide (*n*Bu_4_NBr) was used as a co-catalyst. The reaction conditions are so executed to obtain the optimal reaction yield. The results obtained for catalytic reactions are summarized in Table 1. By employing 10 mg of FCCF and 2 mg of co-catalyst at ambient (1 bar) CO_2_ pressure, a 47% conversion of 1.0 mL ECH to its cyclic carbonate is achieved in 3 hours, as shown in entry 1 of Table 1. However, 28 and 1.8% conversions were observed under the same reaction conditions by separately using either FCCF or the co-catalyst (entries 2 and 3, Table 1), respectively. This trend shows the synergic effect of both FCCF and the co-catalyst in an efficient conversion process. The reaction progress is interesting and >50% conversion is obtained within 4 hours (entry 4, Table 1), and 67 to 87% reaction yields are observed in 5 to 6 hours (entries 5 and 6, Table 1). Complete conversion of epichlorohydrin to its corresponding cyclic carbonate is achieved in 8 hours under the prescribed temperature (100 °C) and CO_2_ pressure (1 bar) (Table 1), with a selectivity of 100% (entry 7, Table 1 and Fig. S2). The recovered catalyst was used in five consecutive catalytic reactions (entries 8–12, Table 1, and Fig. S7). A minute loss of about 0.5 mg of the FCCF (catalyst) is observed during the solid-phase recovery in each catalytic run, with conversions of 98–87% (entries 8–12 in Table 1) without any considerable compromise on selectivity, which shows the efficiency of FCCF as a promising catalyst, which was also supported by the findings of gas phase DFT simulations.

**Table 1:** Cycloaddition of carbon dioxide into epichlorohydrin by the FCCF under specific reaction conditions at 100 °C and ambient CO_2_ pressure (1 bar) without any solvent^a^

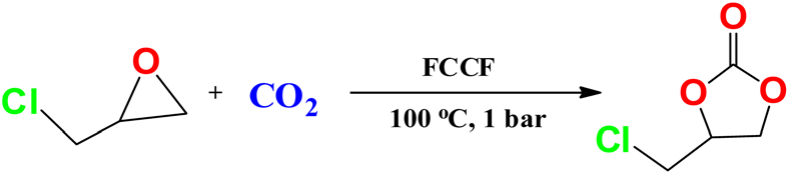					
Entry	Catalyst (mg)	Co-catalyst (mg)	Time (h)	Selectivity^b^ (%)	Conversion^b^ (%)
1	10	2	3	99	47
2	0	2	3	99	1.8
3	10	0	3	99	28
4	10	2	4	99	55
5	10	2	5	99	67
6	10	2	6	99	87
7	10	2	8	100	100
8^i^	10	2	8	99	98
9^^ii^^	9.5	2	8	99	95
10^^iii^^	8.9	2	8	99	92
11^^iv^^	8.3	2	8	99	89
12^v^	7.9	2	8	99	87

a Reaction conditions: epichlorohydrin (13 mmol), FCCF/catalyst (10 mg), and *n*Bu_4_NBr/co-catalyst (2 mg). Catalytic recycling was performed up to five cycles^i–v^ (during recycling, ∼0.5 mg of the catalyst was lost each time).

b Catalytic conversion and selectivity were determined by gas chromatography.

The structural stability of FCCF was observed by taking post catalytic FTIR and PXRD (Fig. S5 and S6, respectively). The FTIR peaks in the spectra of as-synthesized and used (in catalytic cycles) FCCF closely resemble each other, showing that the structure of the catalyst remains intact and stable under the applied catalytic conditions. The powder X-ray diffraction (PXRD) of recycled FCCF showed multiple sharp peaks illustrating the persistence of crystallinity. The catalytic latent of FCCF was also investigated for cycloaddition of propylene oxide (PO) and cyclohexene oxide (CHO) to carbon dioxide (CO_2_) for construction of the respective organic cyclic carbonates, under the opted reaction conditions. The obtained results of catalytic reactions for these epoxides (PO and CHO) are summarized in Table 2. The significant conversions of 42% for propylene oxide and 54% for cyclohexene oxide to respective organic cyclic carbonates (entries 2 and 3, Table 2) with decent selectivity (>95%) when compared to epichlorohydrin (entry 1, Table 2) indicate that FCCF is an appreciably efficient catalyst for the fixation of CO_2_ to different epoxides. The mass spectra are shown in the SI (Fig. S3 and S4).

**Table 2:** Cycloaddition of carbon dioxide into propylene oxide and cyclohexene oxide by the FCCF under specific reaction conditions at 100 °C and ambient CO_2_ pressure (1 bar) without any solvent^a^

Entry	Substrate	Catalyst (mg)	Co-catalyst (mg)	Time (h)	Selectivity^b^ (%)	Conversion^b^ (%)
1	ECH	10	2	8	100	100
2	PO	10	2	8	>95	42
3	CHO	10	2	8	>96	54

a Reaction conditions: epichlorohydrin (ECH: 13 mmol), propylene oxide (PO: 14 mmol) and cyclohexene oxide (CHO: 10 mmol), FCCF/catalyst (10 mg), and *n*Bu_4_NBr/co-catalyst (2 mg).

b Catalytic conversion and selectivity were determined by gas chromatography.

**Fig. 8:**
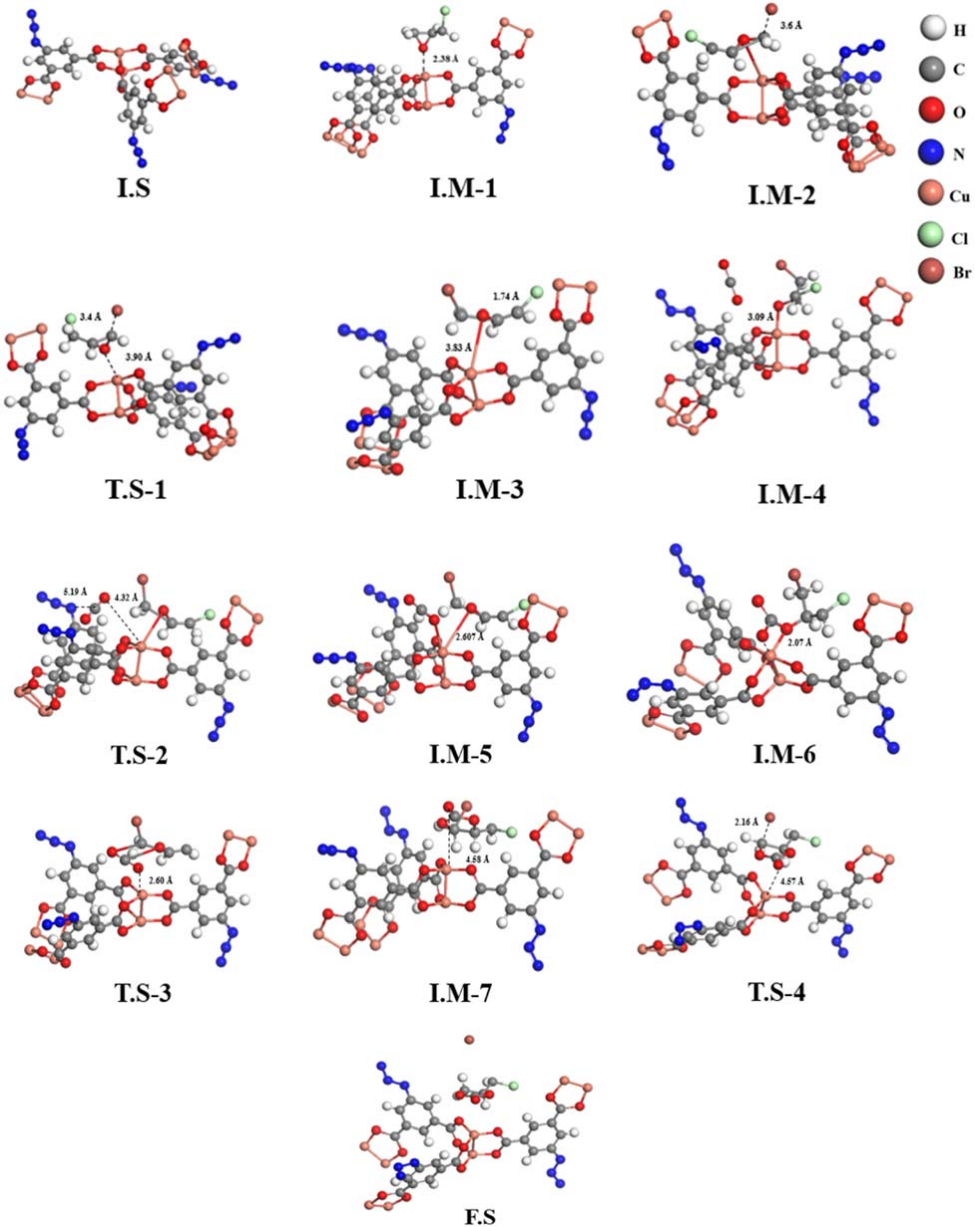
Optimized and transition-state geometries of the various steps involved in the FCCF catalyzed cycloaddition of ECH and CO_2_.

**Fig. 9:**
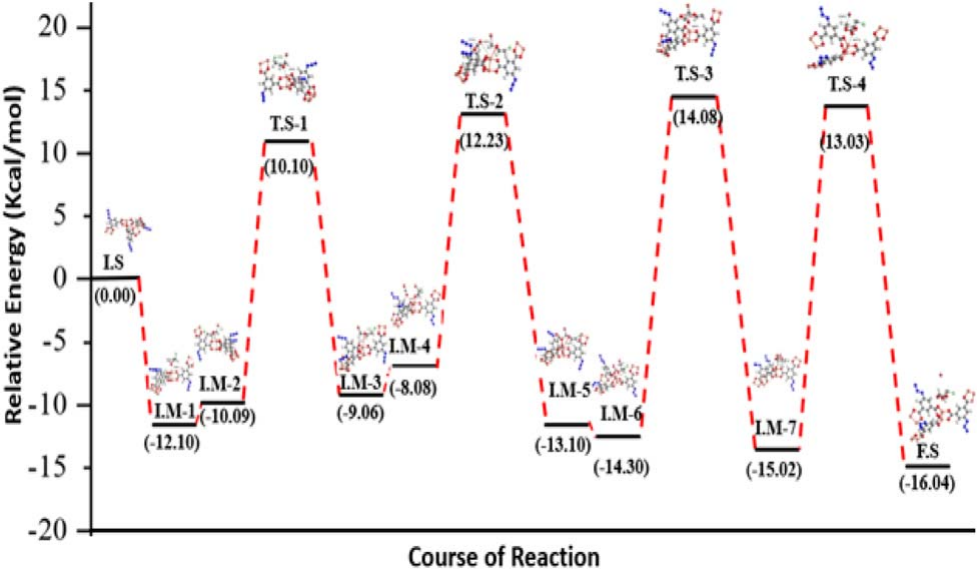
Relative energy diagram of the FCCF-catalyzed cycloaddition of ECH and CO_2_.

The simulated structures of FCCF, epichlorohydrin (ECH), Br^−^ and CO_2_ and optimized initial states, intermediates and transition states are represented in Fig. 8, while the relative energies of different reaction steps are represented in Fig. 9. An overview of the optimized geometry and corresponding electrostatic potential map of FCCF (initial stage/I.S in Fig. 8) and ECH & CO_2_ are presented in Fig. S8a. To reduce the computation cost and show a clearer structure, a unit cell of FCCF is geometrically optimized by performing full geometry relaxation, which shows the zero-point energy state of the optimized catalyst (Fig. 8). The geometry optimization showed that the O atom of the ECH interacted with the Cu atom of the Cu-MOF, forming a Cu⋯O interaction by a distance of 2.38 Å, as shown in I.M-1 (Fig. 8). Reaction energy obtained for this step is −12.10 kcal mol^−1^. In intermediate I.M-2, the Br^−^ of tetrabutyl ammonium bromide (*n*Bu_4_NBr) attacked the methylene site (–CH_2_) of ECH with an optimum distance of 3.6 Å, and formation of the Cu–O bond occurred. Hirshfeld charge density showed that 0.23 e was transferred from the O atom to the d orbital of the Cu atom of FCCF, which also provides an endorsement to the information gained from the HS surface analysis of FCCF about the availability of convenient electropositive sites in the structure of the synthesized MOF for aimed catalytic application. The attack of Br^−^ induces angle strain in the structure, which can result in an increase in relative energy up to −10.09 kcal mol^−1^, as shown in I.M-2. The reaction moves in the forward direction with the ring opening of ECH and formation of I.M-3 by passing through an energy barrier of 10.10 kcal mol^−1^ in transition state T.S-1. The energy obtained in formation of I.M-3 is −9.06 kcal mol^−1^. The formation of I.M-4 occurred with an optimum distance of 3.09 Å between the catalyst (FCCF) and the opened ring of ECH, producing an energy of −8.08 kcal mol^−1^. In the formation of intermediate I.M-5 during the catalytic cycle, O atom of CO_2_ interacted with the Cu center of FCCF, and the azide group (–N_3_) in the bridging linker attacked the partially positive C atom of CO_2_, as shown in transition state T.S-2, which requires a small energy barrier of 12.23 kcal mol^−1^ (Fig. 9). The energy obtained for I.M-5 is −13.10 kcal mol^−1^. The intermediate I.M-5 transformed into I.M-6 with the relative stable energy state of −14.30 kcal mol^−1^, which could be attributed to the adjustment of CO_2_ molecules by the azide (–N_3_) group for capture over the opened ECH ring sites. The reaction proceeded in the forward direction with the formation of a complex at I.M-6 stage, which subsequently resulted in the construction of I.M-7 *via* passing through a transition state T.S-3 (as shown in Fig. 9). The relative energy obtained in the development of I.M-7 is −15.02 kcal mol^−1^. The distance between cyclic oxirene having CO_2_ and catalyst (FCCF) increased from 2.60 Å in T.S-3 to 4.58 Å in I.M-7, showing the potential separation of the targeted cyclic carbonate by rupture of the ring complex in terminal phase of reaction. In the final step (F.S), the Br^−^ ion moved away from the cyclic complex with a distance of 2.16 Å, as shown in T.S-4, leaving behind the catalyst (FCCF) for the next catalytic cycle, along with formation of the targeted product at a relative energy of −16.04 kcal mol^−1^.

The obtained DFT results showed a mechanistic pathway for the effective conversion of ECH to its organic cyclic carbonate. Moreover, the lower energy value of −16.04 kcal mol^−1^ on the relative energy profile obtained by DFT simulations for 100% cyclic carbonate of ECH (entry 7, Table 1) indicates the stability of product as well as the catalytic efficiency of FCCF under the provided catalytic conditions, which offers a quantitative endorsement to the designed experimental and theoretical (DFT) models.

**Fig. 10:**
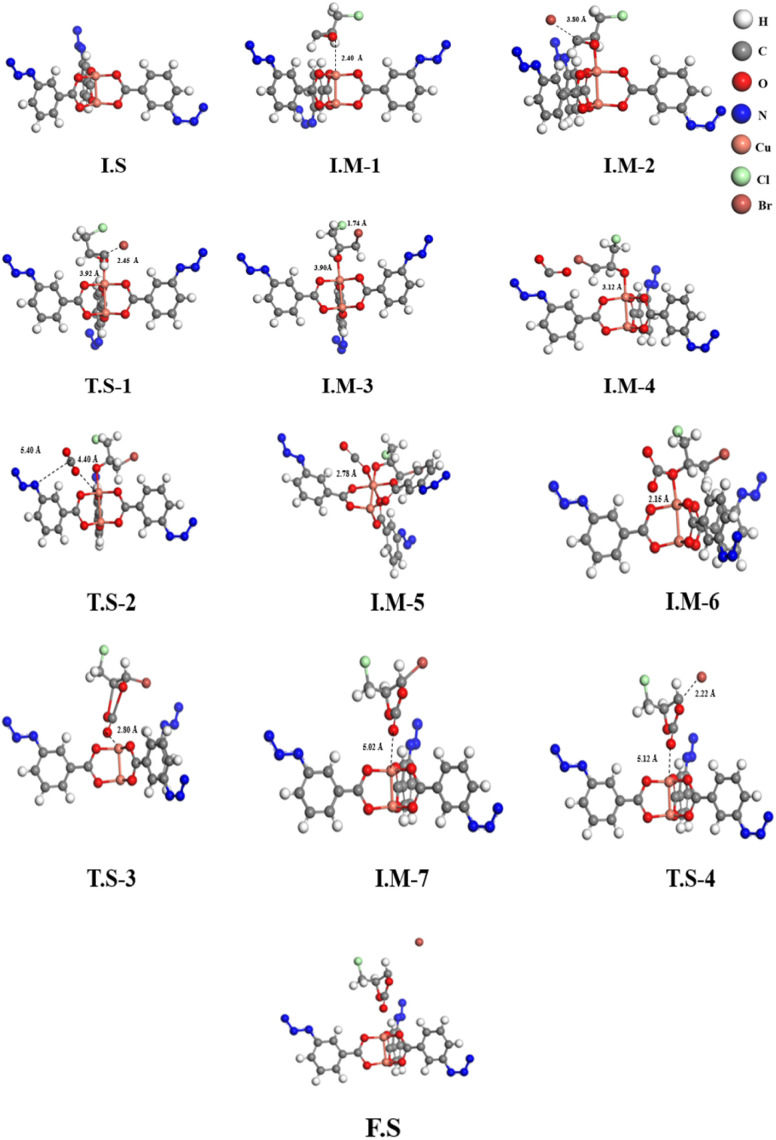
Optimized and transition-state geometries of the various steps involved in the copper(ii)-3-azidobenzoate (CuN_3_B)-catalyzed cycloaddition of ECH and CO_2_.

**Fig. 11:**
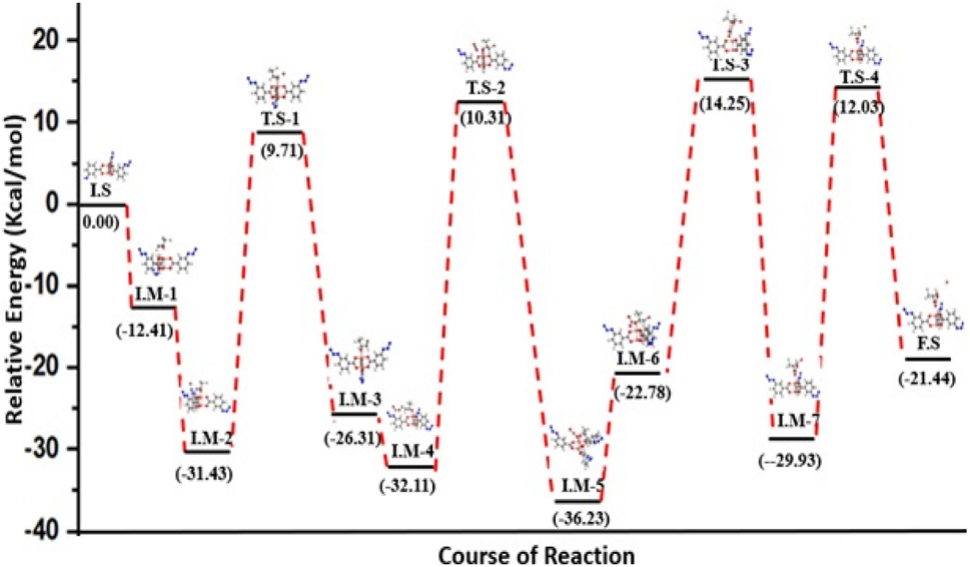
Relative energy diagram of the copper(ii)-3-azidobenzoate (CuN_3_B)-catalysed cycloaddition of ECH and CO_2_.

To better comprehend the involvement of the azide functional group interaction during the catalytic cycle, the same DFT protocol was applied to the simplest case of copper(ii)-3-azidobenzoate (CuN_3_B). This further reduced the computation time/effort and showed the simplest structure. Therefore, we only selected the main interactive section of the FCCF for simulations, while the edge Cu_2_O_2_ units were removed from the catalyst's structure, and the simulated structure of CuN_3_B was optimized in Fig. S8b, also shown as an initial state, *i.e.*, I.S-1 in Fig. 10. Similarly, the respective intermediates and transition states are also represented in Fig. 10, and the relative energies of different reaction steps are represented in Fig. 11. The geometry optimization showed that the O atom of the ECH interacted with the Cu atom of the CuN_3_B, forming a Cu⋯O interaction with a distance of 2.40 Å, as shown in I.M-1 (Fig. 10). In I.M-2, the Br^−^ of the co-catalyst moved closer to the C atom of the ECH (Fig. 10), and a relative energy of −31.43 kcal ml^−1^ was obtained. The binding distance for the Br–C bond reduced from 3.80 to 1.74 Å as the Br atom strongly attached to the C atom of ECH (Fig. 10). The formation of I.M-3 occurred *via* passing through a transition state T.S-1, with a small energy barrier of 9.71 kcal mol^−1^, which reflects the faster reaction occurrence. The reaction energy obtained for this step is −26.21 kcal mol^−1^. In the next step, the CO_2_ molecule moved closer to the ECH ring structure and subsequently interacted weakly with the copper atom of the catalyst, while the azide (–N_3_) group of ligands in CuN_3_B interacted with the C atom of CO_2_, as shown in T.S-2 (Fig. 10), reflecting this site of the linker as an encouraging factor in driving the reaction at a faster rate. The reaction proceeds in the forward direction, and the CO_2_ molecule interacts chemically with the Cu atom of CuN_3_B and forms a chemical bond (Cu–O) with a binding distance of 2.78 Å, as shown in I.M-5 (Fig. 10). This step passes through a small energy barrier of 10.31 kcal mol^−1^, as shown in T.S-2 (Fig. 10). The reaction moved in the forward direction and CO_2_ interacted with the ECH ring structure, forming a complex as shown in I.M-6 (Fig. 10), with a reaction energy of −22.78 kcal mol^−1^. In the next steps of the reaction, O–C bond construction occurred and the Br–C bond slowly ruptured with the ring closer, along with the separation of the attached C=O–Cu bond, having a distance of 5.02 Å in formation phase of the final product, as shown in I.M-7 in Fig. 10. This step passes through a transition state (T.S-3) with a barrier energy of 14.25 kcal mol^−1^, while the reaction energy computed for this step is −29.93 kcal mol^−1^. Finally, the Cu–O=C bond ruptured and the cyclic carbonate of ECH was formed with a relative energy of −21.44 kcal mol^−1^. This lower energy level at the release time of cyclic carbonate of ECH depicts the stability of the obtained product. The results of DFT simulations for FCCF and CuN_3_B show that the azide group plays a pivotal role in catalytic productivity for the formation of the targeted cyclic carbonate of ECH.

The proposed catalytic mechanism for the fixation of carbon dioxide with epichlorohydrin catalysed by FCCF is shown in Fig. S9. The computational calculations demonstrated that oxygen of epichlorohydrin attacks the Lewis acidic site of Cu(ii) and subsequently forms a binding. The Br^−^ ion of *n*Bu_4_NBr attacks the partially positive carbon centre of ECH, resulting in the opening of its ring.

The oxygen of carbon dioxide interacts with the Cu(ii) metal centres. Additionally, the azide functional group develops a binding interaction with the partially positive carbon of CO_2_, supporting the formation of a cyclic intermediate, which is subsequently transformed into the targeted cyclic carbonate in the terminal phase, leaving the FCCF available for participation in the next catalytic cycle. The reaction proceeds in the forward direction with the elimination of cyclic carbonate product in final step. The catalyst (FCCF) is engaged in the next step for another catalytic cycle.

Moreover, the catalytic aptitude of the synthesized FCCF for cycloaddition of epichlorohydrin and carbon dioxide was compared with the literature-reported copper carboxylate MOFs having different functional groups. The comparative catalytic results and applied conditions are summarized in Table 3. The catalytic performance of FCCF was also compared to previously reported copper(ii)-based carboxylate MOFs having different functional groups, as shown in Table 3. Catalysts that showed competitive performance may be due to the use of large catalyst loading, high temperature, longer reaction time, and sometimes even high pressure up to 50 mg, 120 °C, 24 h, and 12 bar, respectively.^3,26,43–47^ Therefore, in this scenario FCCF is a competent copper(ii)-based functionalized carboxylate MOF catalyst for the chemical fixation of carbon dioxide to epoxides for the synthesis of cyclic carbonates.

**Table 3:** Comparative study of the cycloaddition of epichlorohydrin (ECH) to CO_2_ catalyzed by the FCCF and previously reported copper carboxylate MOFs with various functional groups

Catalyst^a^	Ligand's functional groups	Co-catalyst/TBAB (mol%/mmol or mg)	Catalyst/FCCF (mol%/mmol or mg)	Substrate/ECH (mmol)	Reaction conditions	Conversion (%)	Selectivity (%)	Ref.
					*T* (°C) *P* (bar) *t* (h)			
Cu-URJC-8	Amino/-NH_2_ & bipyridine/(C_5_H_4_N)_2_	1	1	1	RT^b^ 12 24	90	>99	37
Cu-MOF-74	Hydroxy/–OH	1	1	1	RT 12 24	67	>99	
Cu-JUC-62	Azo/–N=N–	1	1	1	RT 12 24	81	>99	
HNUST-1	Amide/–N(H)C(O)–	1	1	1	RT 12 24	78	>99	
3D Cu(ii)-MOF (1)	Amide/–N(H)C(O)–	58 mg	50 mg	18	120 8 12	12	100	24
[Cu_6_(TATAB)_4_(DABCO)_3_]_*n*_	Triazine/C_3_N_3_-, azanediyl/–N(H)–Ph– & DABCO/-N_2_(C_2_H_4_)_3_	—	0.02	20	80 8 24	93	NA^c^	38
PNU-25 (a Cu-MOF)	4-Pyridyl-2,3-diaza-1,3-butadiene/–C_12_H_10_N_4_–	0.5	1	15	55 1 18	70	>99	26
PNU-25-NH_2_	-do- & amino/–NH_2_	0.5	1	15	55 1 18	92	99	
HNUST-17	Aminopyridine/H_2_NC_5_H_2_N-, benzoyl/-C_6_H_4_- & amide/–C(O)N(H)–	1 mmol	09 mmol	20	80 1 24	>98.5	NA	3
3D Cu(ii)-MOF (2)	Piperazine/–(CH_2_CH_2_N)_2_–	0.2	0.4	17.5	70 1 16	>99	NA	39
[Cu(MTABA)(H_2_O)]_*n*_	Methoxy 1,3,5-triazine/CH_3_O(C_3_N_3_)- & azanediyl/–N(H)–Ph–	0.5	0.1	1.5	RT 1 24	>99	NA	40
{2Cu(L)(A)·3H_2_O}_*n*_	Triazole/-C_2_H_3_N_3_- & naphthyl/-C_10_H_6_-	0.2	1	10	90 1 24	97	NA	41
2D FCCF	Azide/–N_3_	2 mg	10 mg	1	100 1 08	100	100	T.W.^d^

a Cu-URJC-8 contains 2-aminoterephtalate and bipyridine, Cu-MOF-74 has dihydroxyterephthalate, Cu-JUC-62 has azodiisophthalate, HNUST-1 contains bis(3,5-dicarboxyphenyl)terephthalamide, 3D Cu(ii)-MOF (1) contains (5,5′-(([1,1′-biphenyl]-4,4′-dicarbonyl)bis(azanediyl))diisophthalate), [Cu_6_(TATAB)_4_(DABCO)_3_]_*n*_ contains TATAB = 4,4′,4′′-*s*-triazine-1,3,5-triyl-tri-*p*-aminobenzoate and DABCO = 1,4-diazabicyclo[2.2.2]octane, PNU-25 contains 4-pyridyl-2,3-diaza-1,3-butadiene and BDC, PNU-25-NH_2_ contains NH_2_-BDC and 1,4-bis(4-pyridyl)-2,3-diaza-1,3-butadiene, HNUST-17 has 5,5′-(4,4′-(4-aminopyridine-3,5diyl)bis(benzoyl)bis(azanediyl))diisophthalate, 3D Cu(ii)-MOF (2) contains (5,5′-(piperazine-1,4-diyl)diisophthalate), [Cu(MTABA)(H_2_O)]_*n*_ has 4,4′-((6-methoxy-1,3,5-triazine-2,4-diyl)bis(azanediyl))dibenzoate, {2Cu(L)(A)·3H_2_O}_*n*_, has ligands where L = bis(4-(4*H*-1,2,4-triazol-4-yl)phenyl)methane and A = 1,4-naphthalenedicarboxylate.

b Room temperature.

c Not available.

d This work.

## Conclusion

In the current industrial age, the emissions of carbon dioxide pose a potentially hazardous threat to the environment as well as human life. To overcome the carbon dioxide-based pollution, the researchers are concerned with converting carbon dioxide into valuable products. Keeping this in mind, we synthesized a functionalized copper(ii)-carboxylate framework (FCCF) for application as an efficient catalyst in the chemical fixation of CO_2_ with epoxides, primarily epichlorohydrin. The synthesized catalyst showed very promising results (maximum conversion of CO_2_ into the cyclic carbonate of epichlorohydrin in a short span of time). Density functional theory (DFT) calculations also demonstrated rationality with the anticipated experimental results. The current study can be a step forward in exploring more diversified functionalized copper carboxylate frameworks for the conversion of CO_2_ into valuable industrial products.

## Materials and methods

### Chemicals and instruments

Copper nitrate hexahydrate, epichlorohydrin, tetrabutylammonium bromide, *N*,*N*′-dimethylacetamide, ethyl acetate, biphenyl were purchased from Sigma-Aldrich and used without further purification. CO_2_ gas (>99% purity) was purchased from Pakistan Oxygen Pvt. Ltd, Pakistan. Instruments used for synthesis and characterization included a weighing balance (SHIMADZU AW220), hot air oven (Memmert), ALPHA II Compact FT-IR (Bruker) spectrometer equipped with an ATR, X-ray diffractometer D6 PHASER (Bruker), scanning electron microscope JSM-6510 (JEOL), TGA SDT-Q600 (TA Instruments), and GC-MS 7890B, 5977A (Agilent Technologies).

### Synthesis

Functionalized copper(ii)-carboxylate framework (FCCF) was synthesized at room temperature in *N*,*N*′-dimethylacetamide (DMA).^27^ Greenish-blue coloured crystals of MOF (∼40 mg) were obtained after 3 weeks from the reaction media. The synthesized FCCF was characterized by different analytical techniques. FT-IR (from 4000 to 400 cm^−1^, Fig. 1) showed peaks at 3446 (br), 2933 (m), 2105 (s) 1623 (s), 1581 (s), 1506 (s), 1449 (s), 1397 (s), 1371 (s) 1314 (m), 1292 (m), 1263 (m), 1187 (s) 1013 (s), 799 (s), 773 (s), 727 (m), 670 (m) cm^−1^, which confirmed the formation of the MOF, which was further subjected to activation (for removal of trapped solvents) in a hot air oven before being applied in the planned catalytic experimentation.

### Catalysis

A stainless-steel autoclave equipped with an inlet and vent linked to a cylinder of CO_2_ was used for performing catalytic reactions. The autoclave was purged with carbon dioxide to remove trapped air before initiating the heating process. After the completion of predefined reaction time, heating was stopped, and the autoclave was placed in an ice bath for cooling. The excess (unreacted) CO_2_ was exhausted from the vent of the autoclave. The reaction mixture was subjected to centrifugation to recover the catalyst for reuse in the next catalytic cycle. An aliquot of the supernatant was analysed by gas chromatography using biphenyl as an internal standard to determine the catalytic conversion and selectivity. The DFT simulations were conducted to align with the experimental setup, for getting insight into the mechanistic involvement in fixation of CO_2_ into epichlorohydrin.

### Theoretical analysis

In density functional theory (DFT) calculations for CO_2_ fixation to epichlorohydrin catalysed by FCCF, the DMol3 simulation code was used to perform computations. DFT applying the spin-unrestricted option calculations was used for the catalytic process.^42,43^ The structural optimizations as well as different electronic properties calculations were performed *via* the Perdew–Burke–Ernzerhof (PBE) functional within the generalized-gradient approximation (GGA).^44^ The calculations were performed by a double numerical plus polarization (DNP) basis set.^45^ The Hessian simulations were executed using the same level of theory and basis set to confirm the structural stability, and the absence of negative vibrations confirmed the stability of the optimized structures. The basis set cutoff was set to 4.6 Å, and the thermal-smearing parameter was set to 0.005 a.u. for all computations. For van der Waals interactions corrections, Grimme's scheme, such as DFT-D3 empirical, was used.^46,47^ The structure's relaxation was run at a convergence tolerance of 0.001 Ha Å^−1^ for the force, 0.005 Å for the displacement, and 1 × 10^−5^ Ha for the energy. The energies of adsorption (*E*_ad_) of different complex structures were assessed employing the following equation:*E*_ad_ = *E*_complex_ − (*E*_adsorbent_ + *E*_adsorbate_)where *E*_complex_, *E*_adsorbent_, and *E*_adsorbate_ are the total-optimized energies of the epichlorohydrin/CO_2_@FCCF (complex), FCCF (adsorbent) and epichlorohydrin and CO_2_ molecule (adsorbate), respectively. The linear synchronous transit (LST)/quadratic synchronous transit (QST) and nudged-elastic band (NEB) techniques were applied to calculate the energy barriers.

Furthermore, the use of DFT calculations is proposed to offer mechanistic insights that correspond with the experimental findings. The choice of a gas-phase approach is appropriate as it prevents the introduction of artificial solvation effects that were not present in the actual catalytic system. Although implicit solvation models like COSMO and SMD can consider dielectric effects, they might not accurately represent the specific microenvironment within the pores of MOFs. Incorporating explicit solvent modelling would add more complexity without necessarily enhancing mechanistic understanding, especially since the experiment lacks solvents; considering this, the DFT simulations were conducted in the gas phase to align with the experimental setup, where the catalytic CO_2_ fixation reactions were executed without the use of solvents, meaning the process occurred in a neat system. In the applied catalytic model (heterogeneous catalysis), reacting species were CO_2_ (in gas phase) and epichlorohydrin (liquid substrate, which has to be converted into the respective organic cyclic carbonate) catalysed by FCCF and *n*Bu_4_NBr (solid catalyst and co-catalyst, separated at the end of the optimal reaction time). The unreacted CO_2_ (not fixed into epichlorohydrin) was vented off at the end of the reaction, showing that there was no use of any solvent system, and the computed results provided an orientation consistent with the catalytic reactions.

## Author contributions

H. M. Kashif Javaid: conceptualization, methodology, formal analysis, investigation, writing – original draft, review & editing. Zafar A. K. Khattak: review & editing. Rizwan Ashraf: formal analysis & investigation. Adnan Ali Khan: formal analysis & investigation. Yaqoob shah: formal analysis & investigation. Asim Mansha: investigation. Misbah Zia: review & editing. Hussein A. Younus: formal analysis, review & editing. Nazir Ahmad: Supervision, conceptualization, methodology, formal analysis, investigation, writing – original draft, review & editing. All authors have read and agreed to the published version of the manuscript.

## Conflicts of interest

There are no conflicts to declare.

## Supplementary Material

RA-015-D5RA04781A-s001

## Data Availability

The data supporting this article have been included as part of the SI. Supplementary information: characterization results such as Hirshfeld analysis, mass spectra, FTIR, PXRD, catalytic reuse & mechanism, and DFT optimized molecular geometries. See DOI: https://doi.org/10.1039/d5ra04781a.
