# Supplementary material for: 2D functionalized copper(ii)-carboxylate framework for efficient chemical fixation of carbon dioxide: insights from experimental and theoretical analysis
Source: RSC Adv. 2025 Sep 22;15(41):34797–807. doi: 10.1039/d5ra04781a (PMC12452086; doi:10.1039/d5ra04781a)
Supplement: RA-015-D5RA04781A-s001 [file RA-015-D5RA04781A-s001.pdf]

Supplementary Information (SI) for RSC Advances.
This journal is © The Royal Society of Chemistry 2025

Supplementary information

2D functionalized copper(II)-carboxylate framework for efficient chemical fixation of carbon dioxide: insights from experimental and theoretical analysis

H. M. Kashif Javaid,[a] Zafar A. K. Khattak,[d] Rizwan Ashraf,[e] Adnan Ali Khan,[f,j] Yaqoob Shah,[g] Asim Mansha,[h] Misbah Zia,[i] Hussein A. Younus,[b,c*] and Nazir Ahmad [a,c*]

[a] Department of Chemistry, Government College University Lahore, Lahore-54000, Pakistan, dr.nazirahmad@gcu.edu.pk
[b] Department of Chemistry, Faculty of Science, Fayoum University, Fayoum-63514, Egypt, hay00@fayoum.edu.eg
[c] Nanotechnology Research Center, Sultan Qaboos University, P. O. Box 17, 123, Al-Khoud, Oman
[d] Department of Chemistry, University of Buner, Swari, Buner, Pakistan
[e] Department of Chemistry, University of Agriculture Faisalabad, Pakistan
[f] Department of Chemistry, University of Malakand, Chakdara, Pakistan
[g] Department of Applied Sciences, National Textile University, Faisalabad-37610, Pakistan
[h] Department of Chemistry, Government College University Faisalabad, Faisalabad-38000, Pakistan
[i] Department of Chemistry, University of Sargodha, Sargodha-40100, Pakistan
[j] School of Science, Harbin Institute of Technology, Shenzhen-518055, China

Table S1. Percentage (%) contributions of the interactions revealed in FCCF are summarized:

Interactions	Contributions (%)
All···Cu	0.3
All···O	21.1
All···C	19
All···H	39
All···N	20.6
Cu···All	0.3
O···All	26.2
C···All	22.2
H···All	32.2
N···All	19
N···N	1.9
O··O	2
C···C	7.1
O···C	8.5
C···H	8.9
N···C	9.7
O···H	24
N···H	16.5
H···H	11.4
N···O	10.6
Cu···Cu	0.3

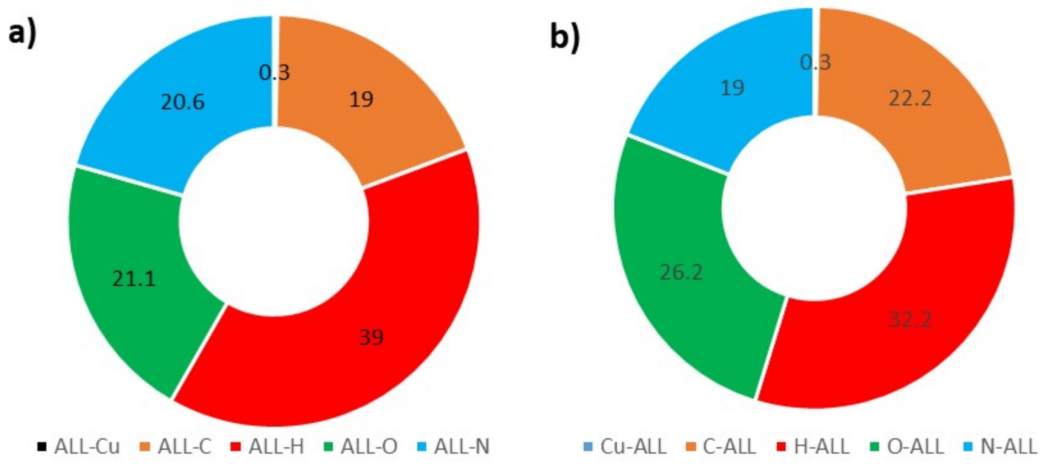

Fig. S1. (a) Percentage contributions of the interaction of an atom present inside the HS to all the atoms present in the surroundings of the HS for FCCF. (b) Percentage contributions of the interaction of all the atoms present inside the HS to an atom outside the HS for FCCF.

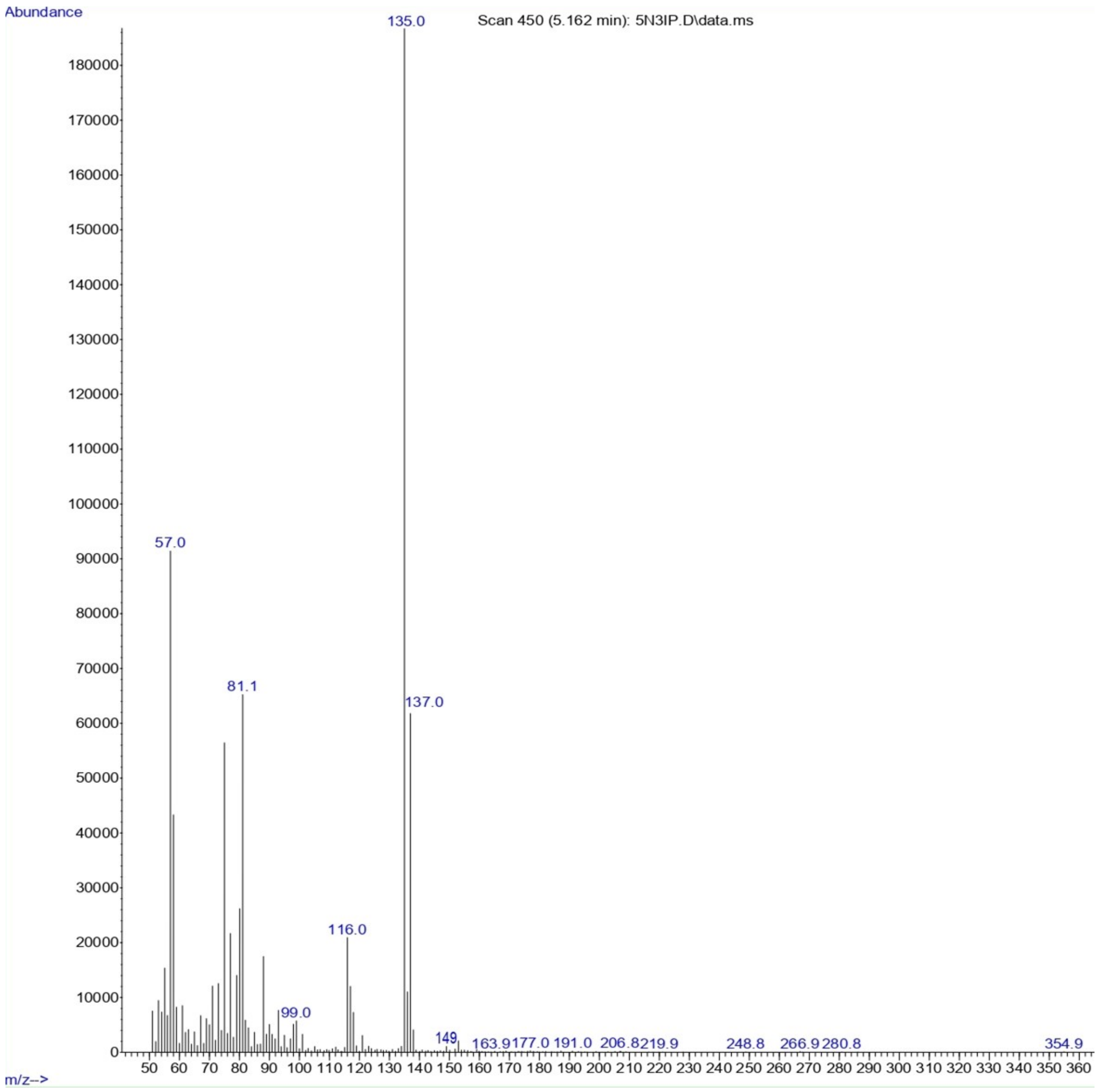

Fig. S2. Mass spectrum of the major product in the reaction mixture obtained from the reaction of epichlorohydrin (ECH) with CO_2 for 8 h catalyzed by FCCF.

The gas chromatographic analysis of the catalytic reaction mixtures revealed interesting results. The observed fragmentation pattern *i.e.* m/z values at 116, 99, 86, 81, 74 and 57 (Fig. S2) corresponds to the possible fragments like -C=O, -Cl, -CH_2Cl, -CHOCl of ECH cyclic carbonate on application of EI-MS scan with further appearance of molecular ion peak at m/z value of 137 along with base peak at 135 in mass spectrum of reaction mixture, obtained from reaction of ECH with CO_2 after 8h catalyzed by FCCF (entry 7, Table 1) corresponds to the successful synthesis of organic cyclic carbonate of epichlorohydrin (ECH).

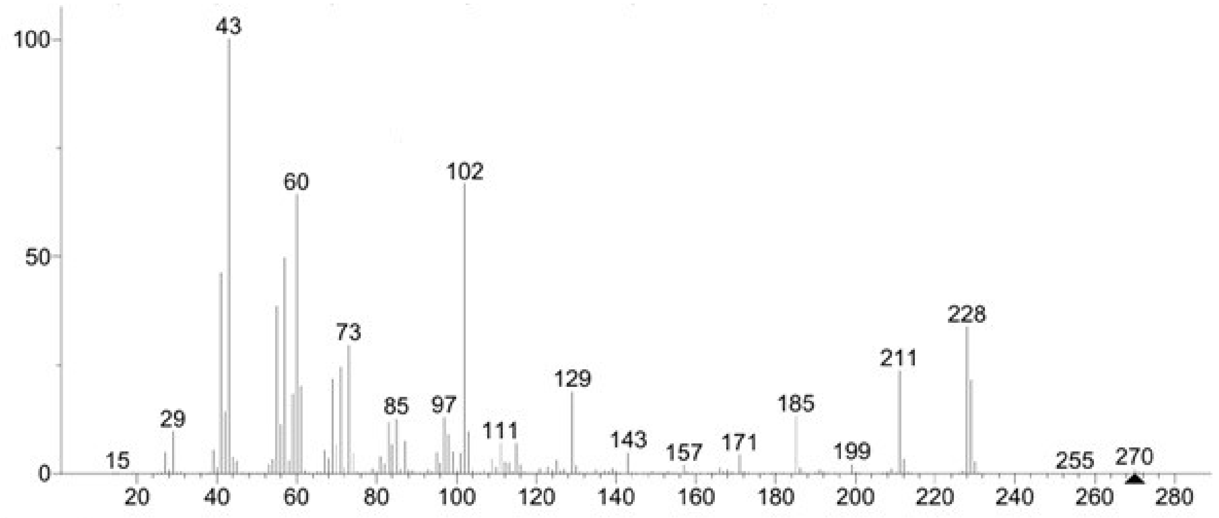

Fig. S3. Mass spectrum of the major product in the reaction mixture obtained from the reaction of propylene oxide (PO) with CO_2 for 8 h catalyzed by FCCF.

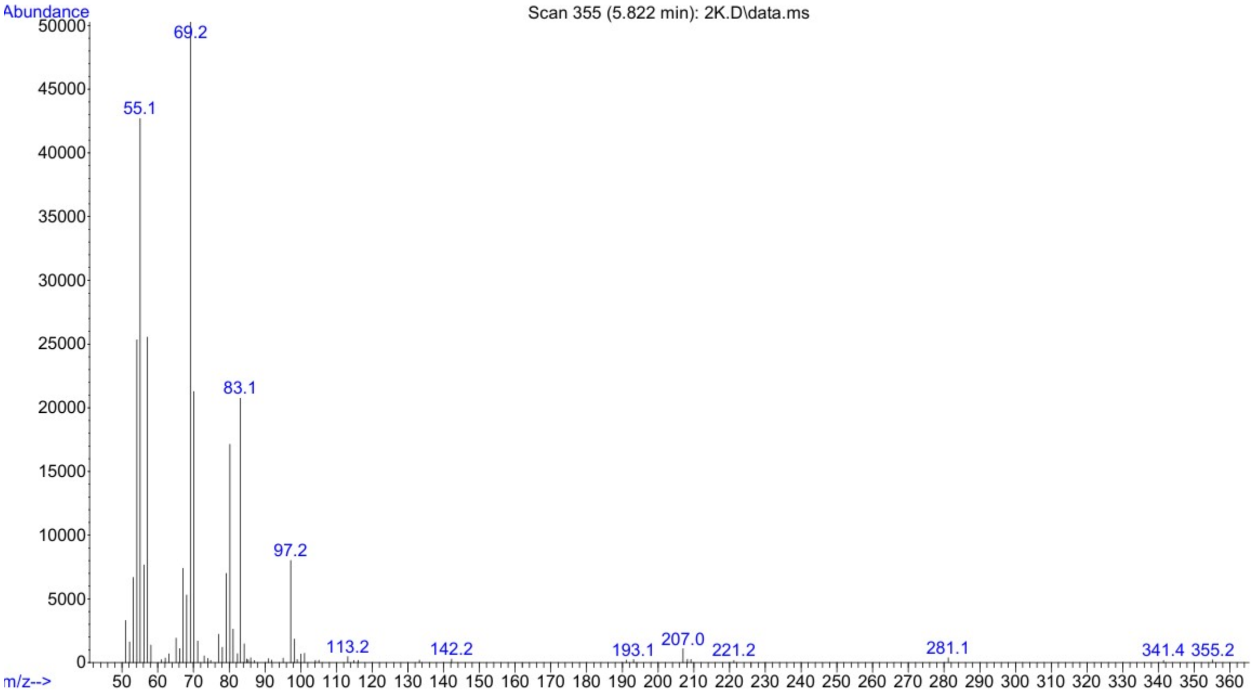

Fig. S4. Mass spectrum of the major product in the reaction mixture obtained from the reaction of cyclohexene oxide (CHO) with CO_2 after 8 h catalyzed by FCCF.

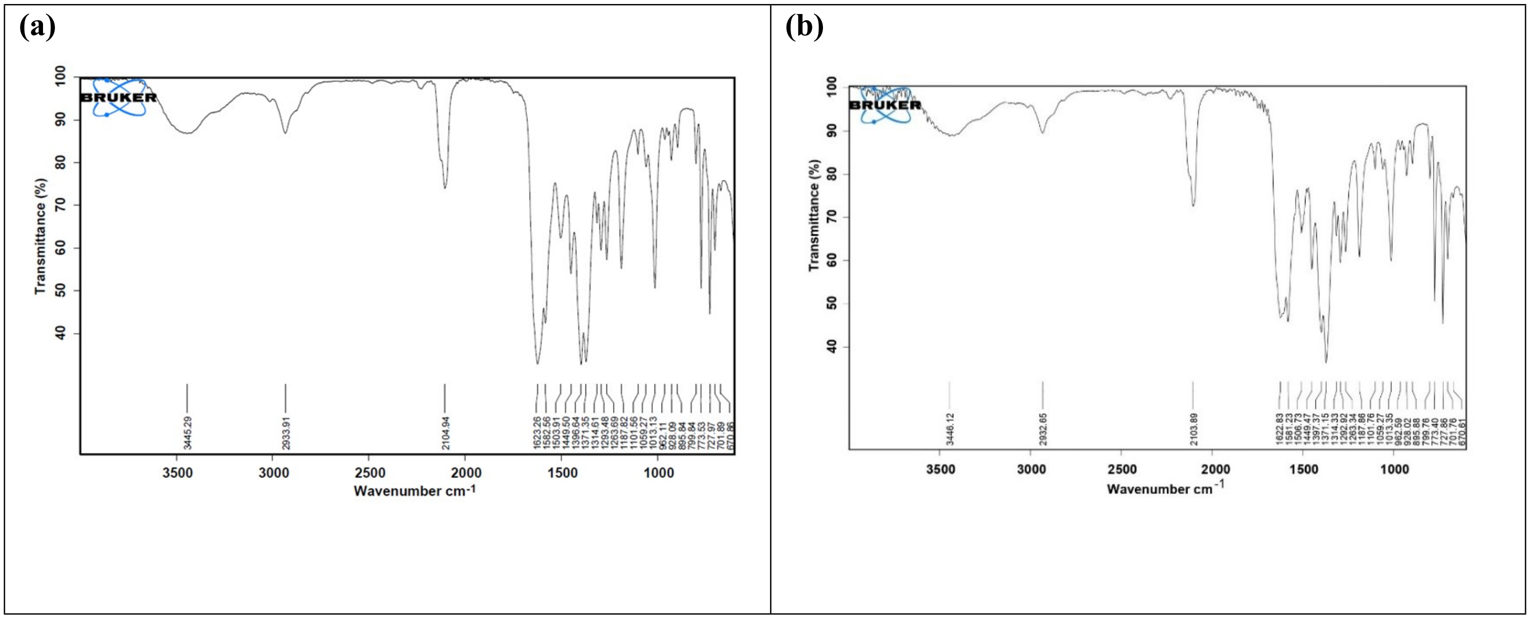

Fig. S5. FTIR Spectrum of FCCF, (a) as-synthesized (b) post catalytic cycles.

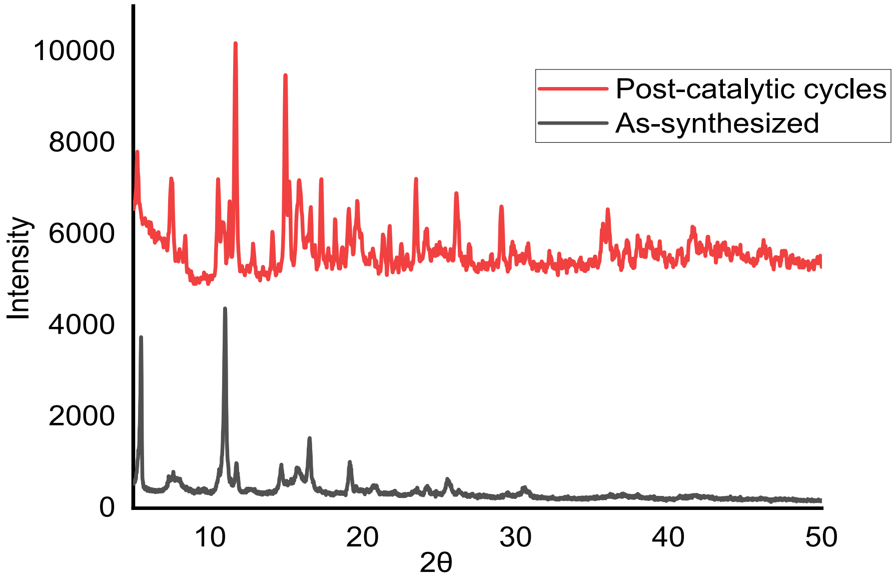

Fig. S6. Powder XRD of FCCF, as synthesized (black) and post catalytic cycles (red).

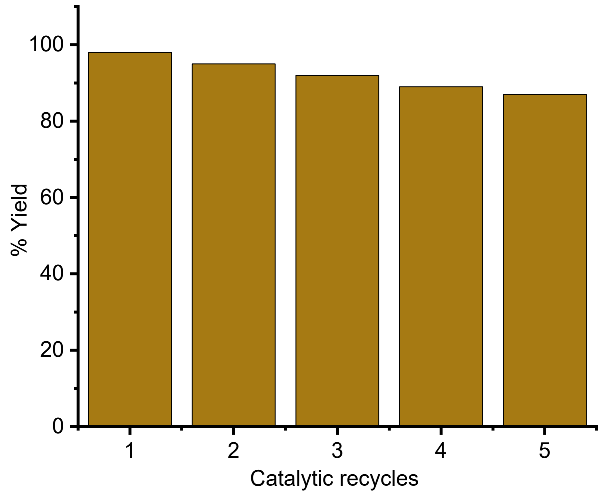

Fig. S7. Percent yield vs catalytic recycles for cycloaddition of CO_2 with epichlorohydrin catalyzed by FCCF.

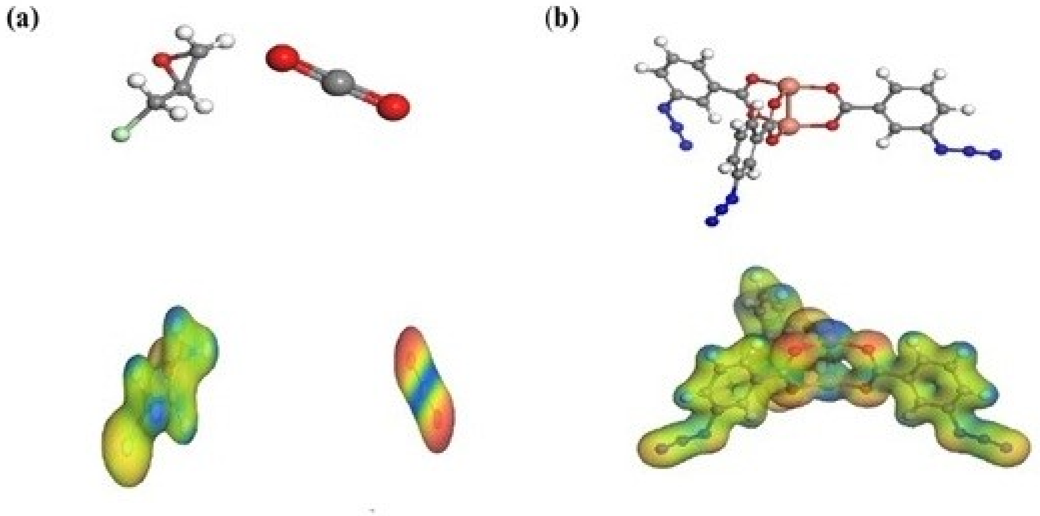

Fig. S8. Optimized geometry and corresponding electrostatic potential map of (a) ECH along with CO_2, and (b) copper(II)-3-azidobenzoate (CuN_3B).

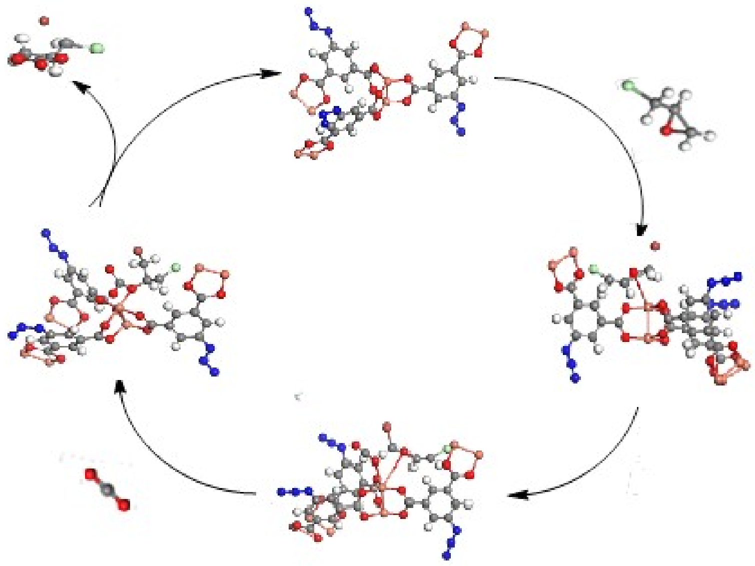

Fig. S9. Proposed catalytic cycle of CO_2 fixation with epichlorohydrin by using FCCF as catalyst.
